# BRD7 inhibits enhancer activity and expression of BIRC2 to suppress tumor growth and metastasis in nasopharyngeal carcinoma

**DOI:** 10.1038/s41419-023-05632-3

**Published:** 2023-02-14

**Authors:** Mengna Li, Yanmei Wei, Yukun Liu, Jianxia Wei, Xiangting Zhou, Yumei Duan, Shipeng Chen, Changning Xue, Yuting Zhan, Lemei Zheng, Hongyu Deng, Faqing Tang, Songqing Fan, Wei Xiong, Guiyuan Li, Ming Tan, Ming Zhou

**Affiliations:** 1 NHC Key Laboratory of Carcinogenesis, Hunan Key Laboratory of Oncotarget Gene, Hunan Cancer Hospital and the Affiliated Cancer Hospital of Xiangya School of Medicine, Central South University, Changsha, 410078 China; 2 Cancer Research Institute and School of Basic Medical Sciences, Central South University, Changsha, 410078 China; 3 The Key Laboratory of Carcinogenesis and Cancer Invasion of the Chinese Ministry of Education, Central South University, Changsha, 410078 China; 4 Department of Pathology, Affiliated Hospital of Jining Medical University, Jining Medical University, Jining, 272100 China; 5 The first clinical college of Changsha Medical University, Changsha, 410219 China; 6 Department of Pathology, the Second Xiangya Hospital, Central South University, Changsha, 410011 China; 7 Graduate Institute of Biomedical Sciences and Research Center for Cancer Biology, China Medical University, Taichung, 406040 Taiwan

**Keywords:** Cancer genomics, Apoptosis

## Abstract

BRD7 functions as a crucial tumor suppressor in numerous malignancies including nasopharyngeal carcinoma (NPC). However, its function and exact mechanisms involved in tumor progression are not well understood. Here, we found that the B7BS was a potential enhancer region of BIRC2, and BRD7 negatively regulated the transcriptional activity and expression of BIRC2 by targeting the activation of the BIRC2 enhancer. Moreover, BIRC2 promoted cell proliferation, migration, invasion as well as xenograft tumor growth and metastasis in vivo, thus functioning as an oncogene in NPC. Furthermore, the recovery of BIRC2 expression could rescue the inhibitory effect of BRD7 on cell proliferation, migration, invasion and xenograft tumor growth and metastasis. In addition, BIRC2 was highly-expressed in NPC tissues, and positively correlated with the TNM stage and negatively correlated with the expression of BRD7. Therefore, these results suggest that BRD7 suppresses tumor growth and metastasis thus functioning as a tumor suppressor at least partially by negatively regulating the enhancer activity and expression of BIRC2, and targeting the BRD7/BIRC2 regulation axis might be a potential strategy for the diagnosis and treatment of NPC.

## Introduction

NPC is an epithelial carcinoma that develops from the nasopharyngeal mucosal lining and occurs at any age in humans [1]. NPC is a multifactorial malignancy associated with both environmental carcinogenic agents, such as Epstein–Barr virus (EBV) infection and genetic factors, such as genetic predisposition [2–4]. However, the molecular mechanisms that induce NPC initiation and promote its malignant progression are still elusive. Despite evident improvements in NPC patient prognosis in recent years [5], most patients are still diagnosed with advanced-stage NPC. Therefore, understanding the mechanisms of NPC carcinogenesis and progression and identifying sensitive biomarkers are of primary importance for the early diagnosis and prognostic evaluation of NPC patients.

BRD7 (bromodomain-containing 7) is a member of the bromodomain-containing protein family, which was first identified in our laboratory in 2000 [6]. Subsequent studies suggest that BRD7 is involved in a wide range of cellular processes, including carcinogenesis [7], glucose metabolism [8], reproduction [9], chromatin remodeling [10], transcriptional regulation and cell cycle progression [11–13]. Accumulating evidences have demonstrated that BRD7 is downregulated in many types of cancers, including nasopharyngeal carcinoma [14, 15], ovarian cancer [16], gastric cancer [17], breast cancer [18] and prostate cancer [19]. Furthermore, BRD7 has been identified as a tumor suppressor gene (TSG) and nuclear transcriptional regulator (NTR) in multiple malignancies [20], in which it participates in the formation of multiple transcription-related complexes as well as the transcriptional regulation process of downstream target genes [21]. However, the mechanism underlying the tumor-suppressive effect of BRD7 as a transcription factor in NPC has not yet been clearly explored. In our previous study, we investigated the chromatin binding sites of BRD7 and its downstream regulatory network by using ChIP-sequencing and digital gene expression profiling (DGE), and the results revealed that BIRC2 is a potential target gene of BRD7 and mediates the regulation of the cell cycle and apoptosis-related pathways [22], these findings might support a potential transcriptional regulation mechanism by which BRD7 functions as a tumor suppressor in NPC.

BIRC2/c-IAP1 is a member of the apoptosis-inhibiting protein family and was identified in a search for TNFR-interacting proteins [23]. BIRC2 is highly conserved in evolution, the N-terminal of BIRC2 contains three baculovirus IAP repeat sequences (BIR domain), and the C-terminal contains the caspase recruitment domain (CARD) and zinc finger domain (Ring domain), the latter of which has an E3 ubiquitin ligase function [24]. Jessy C et al. showed that in addition to its biological function of inhibiting apoptosis, BIRC2 is recruited to the promoter of the CCNE and CCNA genes and directly interacts with the DNA binding domain of the cell cycle-related transcription factor E2F1 to promote transcriptional activation, thereby promoting cell cycle progression [25]. Furthermore, the carcinogenicity of BIRC2 has been demonstrated in a variety of tumors [26], whereas the function of BIRC2 in NPC has rarely been investigated to date.

In this study, we revealed that BRD7 has a motif for binding to intron six of the *BIRC2* gene (chr11:102245603-102246344) (the BRD7 binding site, B7BS), which was further identified as an active enhancer of BIRC2, and BRD7 negatively regulated the transcriptional activity and expression of BIRC2 by targeting the activation of the BIRC2 enhancer region. Moreover, BIRC2 was found to function as an oncogene in NPC, promoting cell proliferation, migration, and invasion as well as xenograft tumor growth and metastasis in vivo, and restoration of BIRC2 expression rescued the inhibitory effect of BRD7 on the malignant phenotype of NPC. Furthermore, we studied the clinical significance of BIRC2 and the association of BIRC2 expression with BRD7 expression in NPC patients. Taken together, our findings proved that BRD7 functions as a tumor suppressor at least partially by inhibiting enhancer activity and the expression of BIRC2 in NPC, and targeting the BRD7/BIRC2 axis might provide a potential strategy for the diagnosis and treatment of NPC.

## Results

### BRD7 inhibits the expression of BIRC2 by negatively regulating the activation of the BIRC2 enhancer

**Fig. 1:**
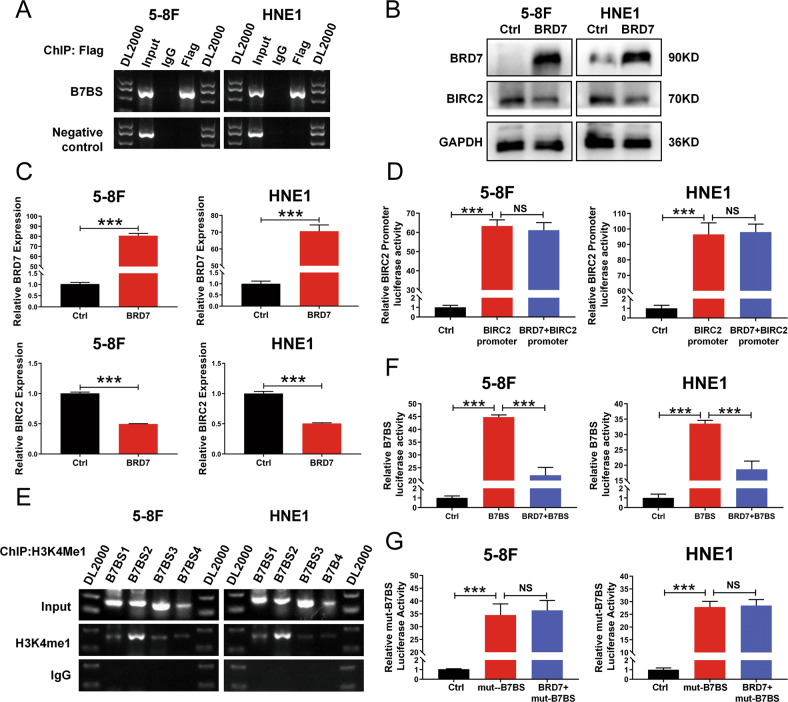
BRD7 negatively regulates the expression of BIRC2 by inhibiting the activation of the BIRC2 enhancer. **A** ChIP-PCR assays using antibody specific for Flag was performed to validate the BRD7-binding sites in the 6th intron of BIRC2, while a 400-bp fragment of the BIRC2 potential promoter region without BRD7 binding site was used as a negative control. **B** Western blotting using antibodies against BRD7 and BIRC2 was performed to confirm BRD7 and BIRC2 protein levels. GAPDH served as an internal control. **C** qRT-PCR assays were used to confirm BRD7 and BIRC2 mRNA levels. GAPDH served as an internal control. **D** The dual-luciferase reporter assays was performed to determine the effect of BRD7 on the promoter activity of BIRC2. **E** ChIP-PCR assay using antibodies specific for H3K4me1 was used to validate the H3K4me1 enrichment at the B7BS, and B7BS was divided into four PCR fragments of about 200 bp, each fragments was amplified with each pair of primers. (**F**) and (**G**) The dual-luciferase reporter assays were used to determine the enhancer activities of B7BS and mut-B7BS and the effect of BRD7 on their activities. The error bars are presented as the mean ± SD. ***P < 0.001; NS, no significance. All experiments were performed in triplicate.

Our previous study demonstrated that BIRC2 was a potential target gene of BRD7 in HEK293 cells and that BRD7 might directly bind to the sixth intron of *BIRC2* [22], which was named as B7BS (BRD7 binding site). To further identify whether there is direct binding between the BRD7 protein and the B7BS region of BIRC2 in NPC cells, the ChIP assay was performed. The results showed that BRD7 could directly bind to the B7BS region of BIRC2 in NPC cells but not to the negative control (Fig. 1A). As BRD7 was identified as a critical nuclear transcription factor, we evaluated the effect of BRD7 on the protein and mRNA expression of BIRC2 by western blotting and qPCR in both 5-8 F and HNE1 cells. The results showed that the overexpression of BRD7 induced a significant decrease in BIRC2 expression at both the protein and mRNA levels (Fig. 1B, C), while knockdown BRD7 increased the protein and mRNA levels of BIRC2 (Fig. S1A, B). However, whether overexpression or knockdown of BIRC2 could not change the expression of BRD7 (Fig. S1C), which provided further evidence that BIRC2 might be a downstream target of BRD7. Therefore, we sought to investigate the transcriptional mechanism by which BRD7 regulates BIRC2 expression. We constructed a luciferase reporter vector containing the potential promoter sequence of BIRC2 predicted by bioinformatics analysis, and the dual-luciferase reporter assay results revealed that the potential BIRC2 promoter exhibited significant luciferase activity but the overexpression of BRD7 had no significant effect on BIRC2 promoter activity (Fig. 1D and Fig. S1D), that is, BRD7 did not affect BIRC2 expression through direct regulation of BIRC2 promoter activity. Then, we sought to determine whether the DNA binding region of BRD7 in BIRC2 is an enhancer or silencer element, and the bioinformatics prediction website (http://genome.ucsc.edu/) revealed that there is an enrichment of the enhancer mark H3K4me1 in the indicated genomic B7BS region (Fig. S1E). Similarly, the results of the ChIP assay were essentially in agreement with this finding, and the 200-386 bp region was found to have higher enrichment of H3K4me1 than the other regions in 5-8 F and HNE1 cells (Fig. 1E), suggesting that the B7BS region might be a potential enhancer element in BIRC2. To verify this result, we constructed a couple of recombinant vectors in which the pGL3 promoter was fused into the B7BS region (the complete sequence of B7BS) and a mutated B7BS (mut-B7BS) respectively, and then performed dual luciferase reporter experiment. As a result, both wild-type B7BS and mut-B7BS regions had significant enhancer activity, and ectopic expression of BRD7 markedly decreased the activity of B7BS, while presented no significant effect on the activity of mut-B7BS (Fig. 1F, G and Fig. S1F), which suggests that B7BS is a critical enhancer element of BIRC2, and the regulation of BRD7 on BIRC2 depended on the binding motif within B7BS in NPC. Therefore, these results demonstrate that BRD7 negatively regulates the transcriptional activity and expression of BIRC2 by targeting the activation of the B7BS region.

### BIRC2 promotes tumor progression and functions as a candidate oncogene in nasopharyngeal carcinoma

**Fig. 2:**
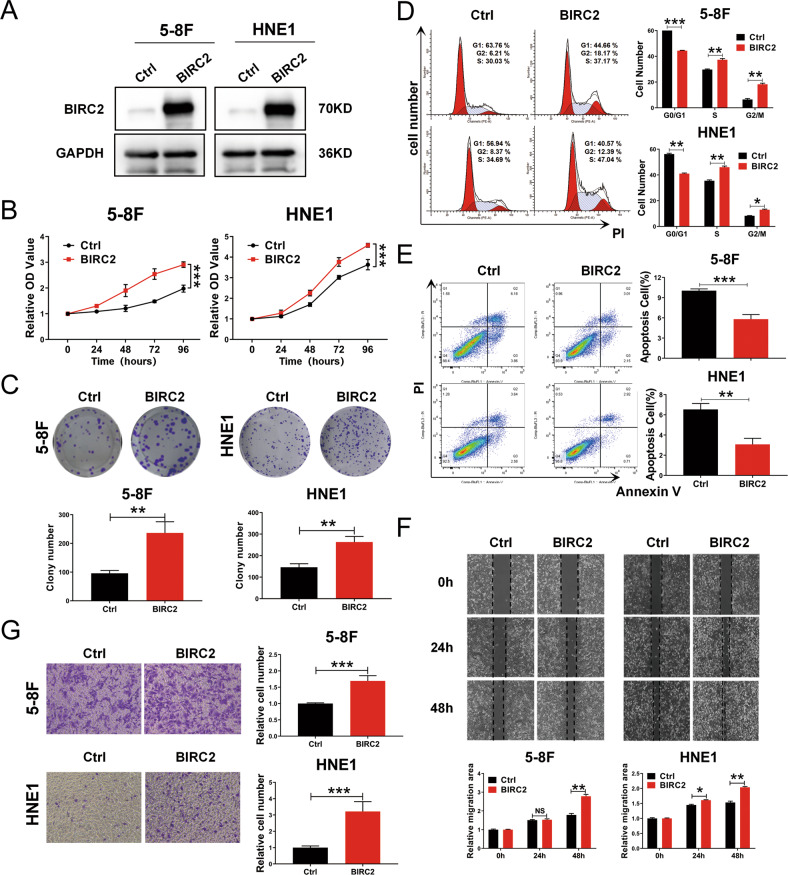
BIRC2 promotes tumor progression and functions as a candidate oncogene in nasopharyngeal carcinoma. **A** Western blotting was performed to detect the BIRC2 protein level using antibody against BIRC2, and GAPDH served as an internal control. **B** CCK-8 assay was performed to detect the cell viability of 5-8 F and HNE1 cells with stable overexpression of BIRC2. **C** Colony-forming assay was used to detect the cell growth of 5-8 F and HNE1 cells with stable overexpression of BIRC2. **D** Flow cytometry analysis of cell cycle distribution, and the representative (left) and statistical results (right) were shown. **E** Flow cytometry analysis of cell apoptosis via Annexin V-FITC and PI double staining, and the representative (left) and statistical results (right) were shown. **F** Scratch wound healing analysis of cell migration in 5-8 F and HNE1 cells stably transfected with BIRC2 expression plasmid or control. **G** Matrigel invasion analysis of cell invasive capabilities in 5-8 F and HNE1 cells stably transfected with BIRC2 expression plasmid or control. Error bars represent the mean ± SD. *P < 0.05, **P < 0.01, ***P < 0.001; NS, no significance. All experiments were performed in triplicate.

**Fig. 3:**
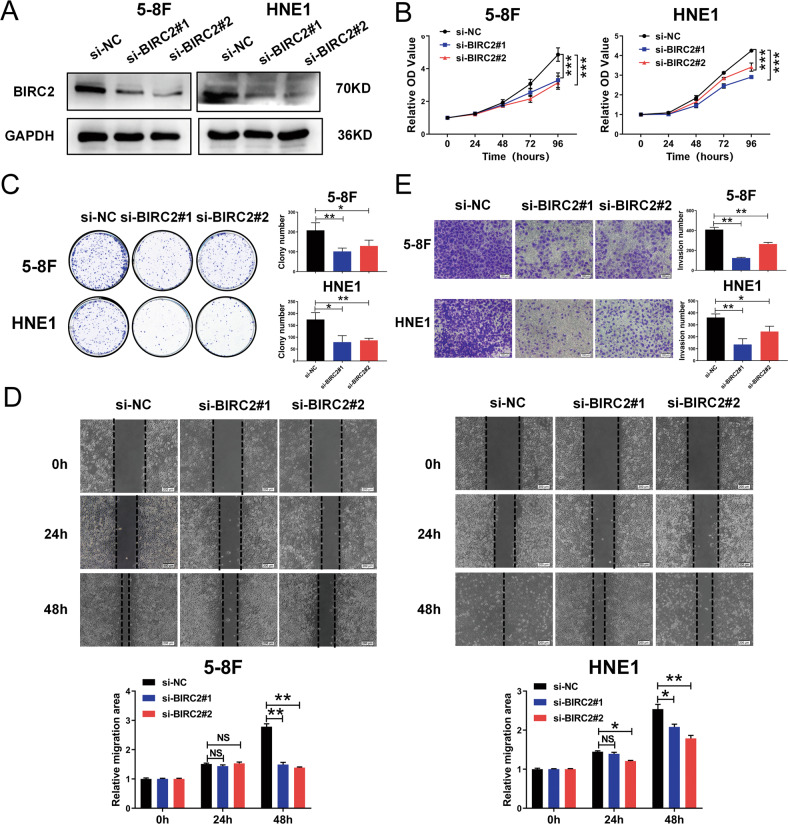
Depletion of BIRC2 by siRNA promotes cell proliferation and metastasis in NPC cell lines. **A** 5-8 F or HNE1 cells were transfected with scrambled siRNA (negative control) and BIRC2 siRNA #1, #2, respectively. Western blotting was used to analyze the silencing efficiency of BIRC2, and GAPDH served as an internal control. (**B**) and (**C**) Cell growth was measured by the CCK-8 assays and colony-forming assays of 5-8 F and HNE1 with transfection of BIRC2 siRNAs or negative control. **D** Cell migration assay was performed with the scratch wound healing analysis. **E** Matrigel invasion analysis of cell invasive capabilities in 5-8 F and HNE1 with transfection of BIRC2 siRNAs or negative control. Error bars represent the mean ± SD. *P < 0.05, **P < 0.01, ***P < 0.001; NS, no significance. All experiments were performed in triplicate.

BIRC2 is a critical member of the c-IAP family and plays pivotal roles in the regulation of nuclear factor-κB (NF-κB) signaling and apoptosis [27, 28]. However, the roles of BIRC2 in NPC remain unknown. We examined the BIRC2 level in a panel of NPC cells and normal nasopharyngeal epithelial NP69 cells and found that the BIRC2 level was markedly increased in NPC cancer cell lines compared with NP69 cells (Fig. S2A). To further confirm the roles of BIRC2 in the malignant progression of NPC, we generated 5-8 F and HNE1 cell lines stably overexpressing BIRC2, and the expression efficiency of exogenous BIRC2 was confirmed by western blotting and qRT–PCR (Fig. 2A and Fig. S2B). Then, we investigated the effect of BIRC2 on cell proliferation by CCK-8 and colony formation assays. As shown in the results, overexpression of BIRC2 significantly enhanced the proliferation and colony-forming capacity of both 5-8 F and HNE1 cells (Fig. 2B, C). To investigate the underlying mechanisms and biological functions of BIRC2, we analyzed the effect of BIRC2 overexpression on cell cycle progression by flow cytometry. We determined and compared the percentage of cells in the S, G0/G1 and G2/M phases and found that overexpression of BIRC2 significantly decreased the number of cells in G0/G1 phase, which was followed by an increase in S-phase cells, in both 5-8 F and HNE1 cells compared with control cells (Fig. 2D). In addition, the effect of BIRC2 on apoptosis was detected by annexin V-FITC/PI double staining and flow cytometric analysis after cells were serum-starved for 24 h, and the results showed significantly lower percentages of apoptotic cells among both 5-8 F and HNE1 cells overexpressing BIRC2 than among the corresponding negative control cells (Fig. 2E). Next, wound healing and transwell assays were performed to investigate the effects of BIRC2 on NPC cells migration and invasion. BIRC2 overexpression obviously promoted the migration and invasion of 5-8 F and HNE1 cells relative to the corresponding negative control cells (Fig. 2F, G). Accordingly, we investigated the effect of BIRC2 knockdown on cell biological functions by RNA interference. We used two different siRNAs targeting BIRC2, and the interference efficiency was confirmed by western blotting and qRT–PCR (Fig. 3A and Fig. S2C). Consistent with the above results, knockdown of BIRC2 inhibits the proliferation, colony formation, migration, and invasion of 5-8 F and HNE1 cells (Fig. 3B–E). Collectively, these results demonstrate that BIRC2 promotes cell proliferation, migration and invasion and therefore plays a potential oncogenic role in NPC cells.

### Restoration of BIRC2 expression reverses the inhibitory effect of BRD7 on cell proliferation

**Fig. 4:**
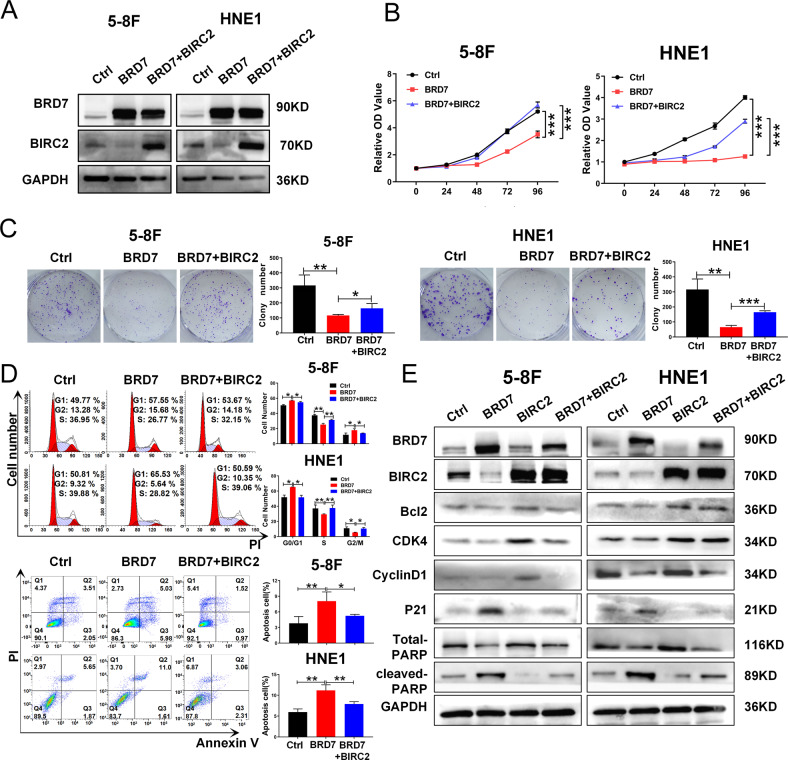
Restoration of BIRC2 expression reverses the inhibitory effect of BRD7 on cell proliferation. **A** Western blotting was performed to confirm BRD7 and BIRC2 protein levels using antibodies against BRD7 and BIRC2. GAPDH served as an internal control. (**B**) and (**C)** CCK-8 analysis and colony-forming assays of cell proliferation in 5-8 F and HNE1 cells stably with BRD7 overexpression, BRD7 and BIRC2 simultaneous overexpression or control group. **D** Cell-cycle analysis and cell apoptosis by flow cytometry. **E** Significantly differently expressed proteins involved in cell cycle progression (P21, CCND1 and CDK4) and survival (Total PARP, Cleaved PARP and Bcl2) in BRD7 overexpression, BIRC2 overexpression and BIRC2 restoration cells, respectively. GAPDH served as an internal control. Error bars represent the mean ± SD. *P < 0.05, **P < 0.01, ***P < 0.001; NS, no significance. All experiments were performed in triplicate.

As BRD7 has been identified as a tumor suppressor that inhibits cell proliferation and tumor growth, which was also confirmed with overexpression and knockdown of BRD7 by CCK-8 and colony formation assays (Fig. S3A, B), while BIRC2 could be negatively regulated by BRD7 and function as an oncogene in NPC, we sought to further ascertain the exact effect of BIRC2 on BRD7-mediated proliferation inhibition in nasopharyngeal carcinoma cells. Therefore, we generated 5-8 F and HNE1 cell lines co-expressing BRD7 and BIRC2 using the BRD7-overexpressing cells (Fig. 4A and Fig. S4). CCK-8 and colony formation assays were performed to detect the effect of BIRC2 on BRD7-mediated cell proliferation inhibition. Consistent with the previous findings, BRD7 had the biological functions of inhibiting cell proliferation and colony formation, while the restoration of BIRC2 remarkably reversed the inhibitory effect of BRD7 on cell proliferation and colony formation (Fig. 4B, C). Next, flow cytometric analysis was used to investigate the roles of BIRC2 in BRD7-mediated cell cycle progression and apoptosis in NPC, and the results showed that BRD7 induced G1/S arrest and induced apoptosis as expected, while the restoration of BIRC2 significantly reversed the effects of BRD7 on the inhibition of cell cycle progression and apoptosis promotion (Fig. 4D).

To further confirm the roles of BIRC2 in BRD7-mediated cell proliferation, cell cycle progression and apoptosis, we detected the expression of cell cycle and apoptosis-related molecules by western blotting, and the results showed that BRD7 decreased the abundances of cell cycle markers such as CDK4 and cyclinD1, cell apoptosis marker such as Bcl2, and increased the protein level of P21 as well as the apoptosis marker cleaved PARP in both 5-8 F and HNE1 cells, while overexpression of BIRC2 produced the exact opposite results, indicating the oncogenic function of BIRC2 in NPC. Moreover, the restoration of BIRC2 significantly restored the regulatory effects of BRD7 on cell proliferation and apoptosis-related molecules (Fig. 4E). These results suggest that BRD7 inhibits cell proliferation, G1/S progression and induces apoptosis at least partially by negatively regulating BIRC2 transcriptional activation and expression.

### Restoring the expression of BIRC2 abrogates the inhibitory effect of BRD7 on cell migration, invasion and EMT

**Fig. 5:**
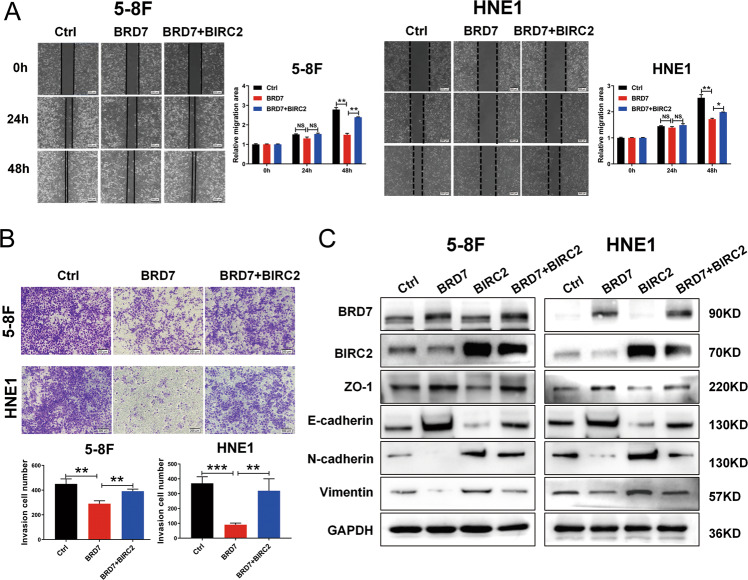
Restoration of BIRC2 expression reverses the inhibitory effect of BRD7 on cell migration and invasion. **A** Scratch wound healing analysis of cell migration in 5-8 F and HNE1 cells stably with BRD7 overexpression, BRD7 and BIRC2 simultaneous overexpression or control. Quantification of the wound recovery rate of the three groups (right). **B** Matrigel invasion analysis of cell invasive capabilities in 5-8 F and HNE1 cells stably with BRD7 overexpression, BRD7 and BIRC2 simultaneous overexpression or control. **C** Significantly differently expressed proteins involved in EMT progression (E-cadherin, N-cadherin, Vimentin, ZO-1) in BRD7 overexpression, BIRC2 overexpression and BIRC2 restoration cells, respectively. GAPDH served as an internal control. Error bars represent the mean ± SD. *P < 0.05, **P < 0.01, ***P < 0.001. All experiments were performed in triplicate.

*BRD7* was also identified as a critical anti-metastatic gene that inhibited cell migration and invasion, which was also confirmed with overexpression and knockdown of BRD7 by transwell assay (Fig. S3C, D). Further experiments were performed to clarify the roles of BIRC2 in BRD7-mediated invasion and metastasis inhibition in NPC cells. Therefore, wound healing and transwell assays were performed to investigate the inhibitory effects of BIRC2 on BRD7-mediated migration and invasion in NPC cells. As expected, overexpression of BRD7 reduced 5-8 F and HNE1 cell migration and invasion potential, while restoration of BIRC2 rescued the cell migration and invasion capabilities (Fig. 5A, B). These results suggest that BRD7 plays an anti-metastatic role in NPC at least partially by negatively regulating BIRC2 transcriptional activation and expression.

Epithelial-mesenchymal transition (EMT), which is a critical cellular process that is often activated during tumor invasion and metastasis, allows epithelial cells to acquire characteristics of mesenchymal cells, such as enhanced motility and invasiveness [29]. EMT is characterized by the loss of epithelial morphology and acquisition of mesenchymal properties, and cell-cell adhesions are weakened, thus leading to increased cell migration and invasion. Our previous study proved that BRD7 inhibited the EMT process and maintains cell integrity in terms of epithelial characteristics and low invasion ability. To further investigate the effect of BIRC2 on the BRD7-mediated EMT process in NPC cells, a series of EMT-related molecules were detected by western blotting. BRD7 overexpression decreased the protein expression of the mesenchymal markers Vimentin and N-cadherin. It increased the protein expression of the epithelial markers E-cadherin and ZO-1. In contrast, overexpression of BIRC2 produced the opposite results, indicating that BIRC2 plays a role in promoting the EMT process in NPC cells. Moreover, the changes in the expression levels of these EMT-related markers were significantly reversed after BIRC2 restoration (Fig. 5C). To observe the changes more intuitively in marker molecule levels in the process of EMT, we detected the influence on the mesenchymal marker Vimentin by immunofluorescence staining. We found that the fluorescence of Vimentin in BRD7-overexpressing cells became weaker than that in negative control cells, while the loss of Vimentin fluorescence was reversed through reactivation of BIRC2 expression (Fig. S5). These results support the hypothesis that BRD7 may inhibit the EMT process by negatively regulating the expression of BIRC2 in vitro NPC cells.

### BRD7 inhibits tumor growth and metastasis in vivo through regulation of BIRC2 expression

**Fig. 6:**
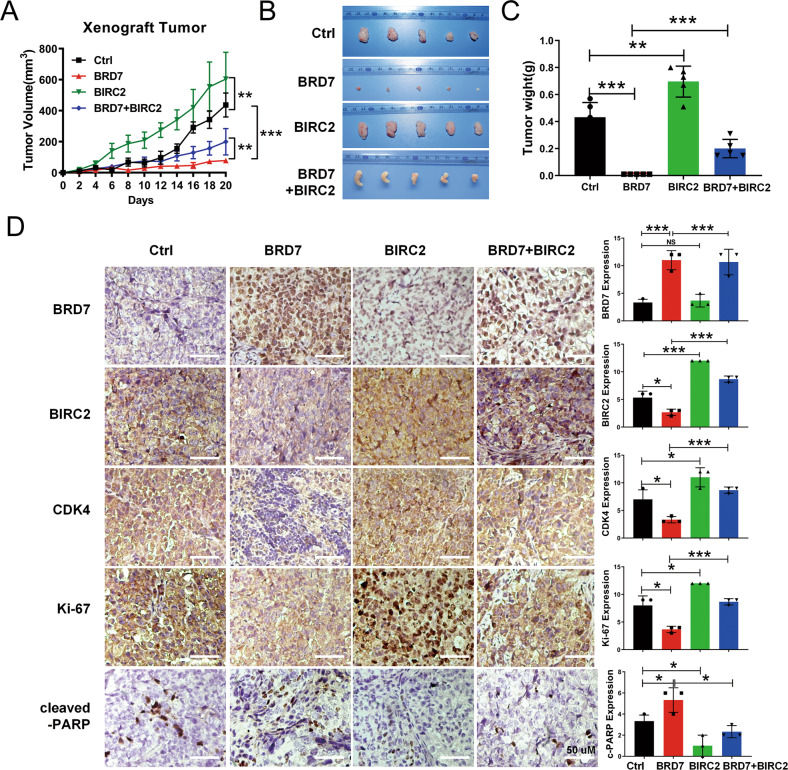
BRD7 inhibits tumor growth in vivo through regulation of BIRC2 expression. **A** Growth curve of tumor xenografts. **B** Representative tumor images of the 5-8 F xenograft model in nude mice. **C** Tumor weight quantification. **D** IHC (DAB staining) for Ki-67, CDK4 and cleaved-PARP in the 5-8 F xenograft model. Three tumors were analyzed per group. Original magnification, 200×; scale bars represent 50 μm. Error bars represent the mean ± SD. *P < 0.05, **P < 0.01, ***P < 0.001.

To ascertain that suppression of BIRC2 expression by BRD7 is responsible for BRD7’s tumor suppressor function in NPC tumorigenesis and tumor progression, we sought to determine whether the restoration of BIRC2 expression reverses the tumor-suppressive roles of BRD7 induced in vivo. Therefore, we conducted in vivo experiments using xenograft mouse models established with a series of 5-8 F cell lines in four groups of mice, i.e., 5-8 F/control, 5-8 F/BRD7, 5-8 F/BIRC2 and 5-8 F/BRD7 plus BIRC2. Next, cells were injected subcutaneously into the flanks of 5-week-old female nude mice; tumors began to grow on the 4-6th day, and the body weight and tumor size were measured once every 2 days. After 20 days, the mice were euthanized and photographed (Fig. S6A), and the subcutaneous tumors were carefully removed, photographed, and weighed. As expected, BIRC2 overexpression increased the tumor growth rate and tumor weight relative to those in the control group, while BRD7 overexpression decreased the growth rate and tumor weight relative to those in the control group, these effects were significantly reversed after the restoration of BIRC2 (Fig. 6A–C). Meanwhile, the expression of BRD7 and BIRC2 in xenograft tumor tissues was confirmed by Western blotting (Fig. S6B). In addition, immunohistochemical (IHC) staining was performed to detect the expression of some critical molecules related to the cell cycle and apoptosis in xenograft tumor tissues. The results showed that BRD7 was successfully overexpressed in xenograft tumor tissues and that the expression of BIRC2 was decreased in the BRD7 overexpression group. Meanwhile, BIRC2 expression was successfully restored in the rescue group. Moreover, IHC staining also revealed that BIRC2 increased the expression of Ki67 and CDK4, and decreased the protein level of cleaved-PARP, consistent with the in vitro experiments, while the opposite results were observed in the BRD7 group, and these changes were significantly reversed as a consequence of BIRC2 restoration (Fig. 6D). Therefore, these results suggest that BRD7 inhibits tumor growth in vivo at least partially by negatively regulating the expression of BIRC2 in NPC cells.

**Fig. 7:**
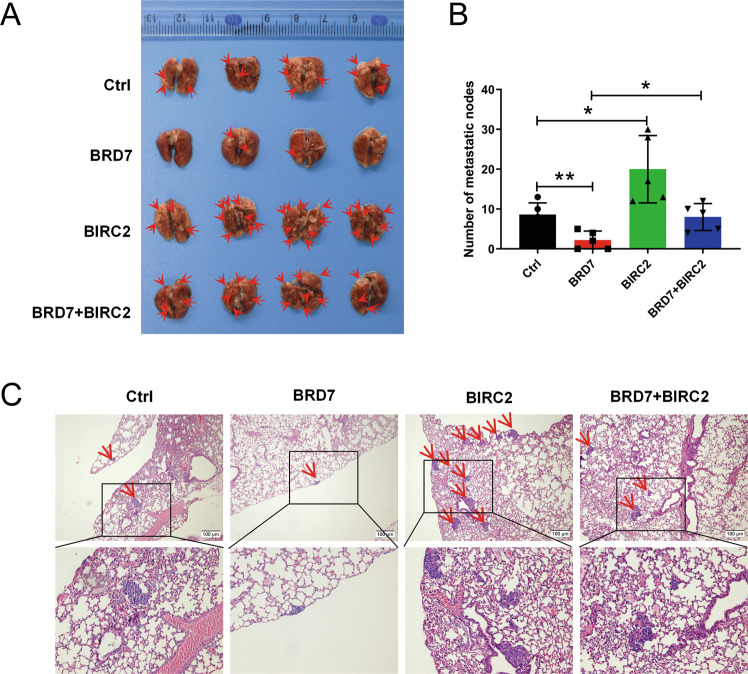
BRD7 inhibits tumor metastasis in vivo through regulation of BIRC2 expression. **A** Representative image of macroscopic mouse lung tissue in the metastatic tumor model. **B** The number of metastatic lung nodules of every mouse per group was counted in microscopy. **C** H&E staining is shown in control, BRD7 overexpression, BIRC2 overexpression and BIRC2 restoration group. Red arrows indicate metastatic tumors, scale bar, 100 μm. Error bars represent the mean ± SD. **P < 0.01, ***P < 0.001.

We next established a lung metastatic colonization model by inoculating the series of 5–8 F cells indicated above into the tail veins of mice. Eight weeks after injection, the mice were sacrificed, and tissues were examined with routine histological processes. Lung colonization and lung metastatic nodule formation were monitored and quantified in the mice. As expected, overexpression of BIRC2 increased the number of lung metastatic nodules. Conversely, overexpression of BRD7 reduced the number of nodules, while restoring the expression of BIRC2 in BRD7-overexpressing cells rescued the suppressive effect of BRD7 on lung metastasis of tumors in the mice (Fig. 7A, B). Furthermore, the lung metastatic nodules in the mice were confirmed by H&E staining of tissue slices. BIRC2 overexpression increased lung metastatic colonization compared with that in the control group, while BRD7 overexpression considerably attenuated lung colonization, whereas restoring BIRC2 expression restored lung metastatic activity (Fig. 7C). No metastatic nodules were detected in the other organs of the mice. Collectively, these in vivo results are consistent with the patterns observed in the in vitro experiments and further confirm that BRD7 inhibits tumor metastasis at least partially by negatively regulating BIRC2 transcriptional activity and expression.

### BRD7 expression is negatively correlated with BIRC2 expression in clinical biopsy tissues

**Fig. 8:**
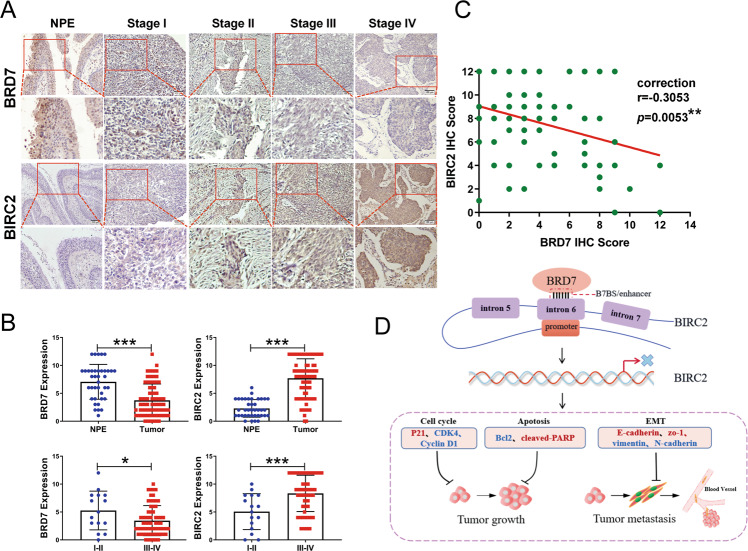
BRD7 expression was negatively correlated with BIRC2 in clinical NP and NPC tissues. **A** Representative image of BRD7 and BIRC2 expression in noncancerous nasopharyngeal tissues and different clinical TNM stages of NPC detected by IHC. **B** Statistical diagram of BRD7 and BIRC2 expression in noncancerous nasopharyngeal tissues and different clinical TNM stages of NPC. **C** Correlation between BRD7 expression and BIRC2 expression. **D** A schematic map showing the tumor-suppressive mechanism of BRD7 that occurs by repressing the enhancer activity and expression of BIRC2 in NPC. Red represents upregulated genes, and blue represents downregulated genes. Error bars represent the mean ± SD. *P < 0.05, **P < 0.01, ***P < 0.001.

Based on the mechanism identified above, we proceeded to explore the expression and clinical relevance of BIRC2 and BRD7 in both NPE and NPC tissue samples. To this end, IHC staining of BIRC2 and BRD7 was performed in tissues derived from 38 healthy donors and 82 patients varying in age, gender, and clinical stage. BIRC2 was highly expressed in NPC tissues compared with noncancerous NP tissues, and its expression in clinical stages III and IV was significantly higher than that in stages I and II. Conversely, BRD7 levels in NPC tissues were lower than in noncancerous NP tissues, and BRD7 expression in clinical stages III and IV was significantly lower than that in stages I and II, consistent with the previous results (Table 1, Fig. 8A, B). Furthermore, there was a significant negative correlation between BRD7 and BIRC2 expression in these tissues (Pearson correlation coefficient (r) = -0.3053, *P* = 0.0053) (Fig. 8C). Taken together, our data indicate that both BRD7 and BIRC2 might be involved in the malignant progression of NPC and targeting the BRD7/BIRC2 axis might be a promising strategy for the clinical diagnosis and treatment of NPC.

**Table 1:** Association between the expression of BRD7, BIRC2 and NPC clinical pathological features (*n* = 82).

	BRD7 expression			BIRC2 expression			BRD7/BIRC2		
Variables Features	Low	High	*p*	Low	High	*p*	L-H	H-L	*p*
Gender									
Male *(n* = 56)	41 (73%)	15 (27%)	0.3992	22 (39%)	34 (61%)	0.2116	30 (54%)	9 (16%)	0.5082
Famale (n = 26)	22 (85%)	4 (15%)		6 (23%)	20 (77%)		20 (77%)	3 (12%)	
Age									
≤47 (*n* = 42)	33 (79%)	9 (21%)	0.7959	17 (40%)	25 (60%)	0.25	25 (60%)	6 (14%)	0.9999
>47 (*n* = 40)	30 (75%)	10 (25%)		11 (27%)	29 (73%)		25 (63%)	6 (15%)	
Tumor size									
I-II (*n* = 52)	40 (77%)	12 (23%)	0.9789	17 (33%)	35 (67%)	0.7147	32 (62%)	8 (15%)	0.7675
III-IV (*n* = 30)	23 (77%)	7 (23%)		11 (37%)	19 (63%)		16 (53%)	5(10%)	
Clinical stages									
I-II (*n* = 16)	9 (56%)	7 (44%)	0.0458*	10 (63%)	6 (37%)	0.0163*	7 (44%)	7 (44%)	0.003**
III-IV (*n* = 66)	54 (82%)	12 (18%)		18 (27%)	48 (73%)		43 (65%)	5 (8%)	

The following abbreviations were used: *H* high expression, *L* low expression. Statistical analysis was performed using the Chi-squared test. *P <0.05; **P <0.01.

## Discussion

BRD7 was identified as a critical nuclear transcriptional regulatory factor [13]. In addition, BRD7 interacts with p53 and is required for p53-dependent replication- or oncogene-induced senescence [30], and BRD7 regulates BRCA1-dependent transcription through its direct interaction with BRCA1 [21]. Our previous results also demonstrated that BRD7 inhibits G1/S progression, induces apoptosis, and reverses malignant behaviors in NPC cells through the Ras/MEK/ERK [31], Rb/E2F [12] and PI3K/AKT signaling pathways [15, 32]. In this study, BIRC2 was identified as a direct target of BRD7, which functions as a transcriptional regulatory factor to inhibit carcinogenesis and tumor progression at least partially by negatively regulating the enhancer activation and expression of BIRC2 in NPC.

Previous investigations revealed that BRD7 could act as a transcriptional regulator and transcriptionally downregulate miR-141 by repressing its promoter activity, whereas no apparent binding site of BRD7 was found in the potential promoter region of miR-141 [15]. In addition, BRD7 activates Bak promoter activity and indirectly induces Bak expression [33]. Therefore, the exact mechanism of BRD7 functions as a tumor-suppressor by influencing its target genes has rarely been reported. Cis-regulation of gene expression is primarily mediated by interactions of enhancer/reporter elements with promoters of their target genes. The enhancer is a cis-acting DNA regulatory element that is recruited to a promoter to form an enhancer-promoter loop and increases transcriptional activity with the help of transcriptional regulators independent of their position and orientation [34]. Based on this principle, BRD7 might interfere with the formation of the promoter-enhancer loop of BIRC2, thus inhibiting BIRC2 transcriptional activation. However, the core enhancer region within B7BS and exact mechanism by which BRD7 interferes with the approach of the enhancer to the promoter of BIRC2 needs to be further studied in the future. Although enhancers are often considered to be located in the upstream or downstream of genes [35, 36], they can also be located within the gene or in a noncoding intergenic region. Aleksandra et al. identified an enhancer region in a noncoding intergenic region between the *TP63* and *LEPREL1* genes on chromosome 3q28 that regulated gene expression in cis, and the presence of this enhancer region significantly increased ΔNTP63 promoter activity in bladder cancer cells [37]. Therefore, it can be understood that the BIRC2 enhancer (B7BS) is located in the sixth intron of *BIRC2*. Generally, active enhancers are marked by a combination of H3K4me1- and H3K27ac-enriched domains [38, 39]. In the current study, we found enrichment of the enhancer mark H3K4me1 in the indicated B7BS genomic region. The dual luciferase reporter assay showed that the B7BS region had significant enhancer activity and that overexpression of BRD7 significantly decreased the enhancer activity of B7BS. The above results indicate a direct transcriptional regulatory mechanism of BRD7 as a transcription factor that negatively regulates the expression of BIRC2.

BIRC2/c-IAP1 is a member of the inhibitor of apoptosis (IAP) family of proteins that plays a critical role in suppressing apoptosis and promoting cell cycle progression [40, 41]. Several reports indicate that BIRC2 gene expression is upregulated in multiple types of cancers and that overexpression of BIRC2 counteracts apoptosis and increases the resistance of tumor cells to chemotherapy and immunotherapy [24, 42, 43]. Especially, BIRC2 has been reported as a hypoxia-induced gene that impairs CD8 + T and NK cell-mediated killing of tumor cells [43]. In this study, we found that in addition to its anti-apoptotic function, ectopic expression of BIRC2 can significantly promote cell proliferation, G1/S progression, invasion, EMT and in vivo tumor growth and metastasis, suggesting that BIRC2 functions as an oncogene in nasopharyngeal carcinoma progression. To our knowledge, this is the first report indicating that BIRC2 functions as a multifaceted oncogene in NPC.

BRD7 is involved in NPC tumor development and progression as a tumor suppressor and negatively regulates the expression of BIRC2. In parallel, restoring the expression of BIRC2 in BRD7-overexpressing NPC cells partially reversed the effects of BRD7 on cell proliferation, apoptosis, and metastasis in vitro and in vivo. Considering these findings, we further explored the expression of molecular markers that are involved in cell cycle progression, survival, and EMT, including cyclin D1, CDK4, P21, Bcl2, cleaved PARP, total PARP, Vimentin, N-cadherin, E-cadherin and ZO-1. Accordingly, these molecules appeared to undergo expression changes consistent with the phenotypic changes. Therefore, these results suggest that BRD7 inhibits cell proliferation, migration, and invasion, as well as xenograft tumor growth and metastasis by negatively regulating the expression of BIRC2. Furthermore, to explore the biological functions of BIRC2 in NPC, we experimentally confirmed that BIRC2 is upregulated in NPC biopsy specimens and its expression is positively correlated with the TNM stage and negatively correlated with BRD7 expression. Therefore, these results support the hypothesis that the BRD7/BIRC2 regulatory axis is involved in carcinogenesis and tumor progression and that targeting BRD7 and BIRC2 is a potential strategy for diagnosing and treating NPC.

In conclusion, we confirmed a potential enhancer region in BIRC2 and showed that BRD7 negatively regulates the transcriptional activity and expression of BIRC2 by targeting the activation of the BIRC2 enhancer. Moreover, BIRC2 was identified as an oncogene that promotes cell proliferation, G1/S progression, EMT, invasion, and in vivo tumor growth and metastasis in nasopharyngeal carcinoma. Furthermore, restoration of BIRC2 expression at least partially reversed BRD7-mediated inhibition of malignant behaviors in NPC cells (Fig. 8D). In addition, BIRC2 was upregulated in NPC biopsy specimens, and its expression was positively correlated with the TNM stage and negatively correlated with BRD7 expression. Therefore, these results suggest that BRD7 suppresses tumor growth and metastasis at least partially by negatively regulating the enhancer activity and expression of BIRC2 and that targeting the BRD7/BIRC2 regulatory axis is a potential strategy for the diagnosis and treatment of nasopharyngeal carcinoma.

## Materials and methods

### Clinical specimens

A total of 82 NPC and 38 noncancerous nasopharyngeal tissues with chronic inflammation of the nasopharyngeal mucosa from independent patients aged 23-67 years were collected from 2014 to 2017 at the Second Xiangya Hospital of Central South University (Changsha, China). Written informed consent was obtained from all participants before the study, and the Ethics Review Committee approved all protocols, specimen usage, and data retrieval of the Second Xiangya Hospital of Central South University (Scientific and Research Ethics Committee, No. Y202/2014). All the clinicopathological characteristics information of the NPC patients were obtained, including pathology diagnosis, sex, age, node metastasis, tumor size, distant metastasis and TNM stage. All tissue samples were paraffin-embedded. We reviewed the clinicopathological data, and TNM classification was based on the criteria of the American Joint Committee on Cancer (AJCC, 6th edition).

### Cell culture

The immortalized nasopharyngeal cell line NP69 and human NPC cell lines CNE1, CNE2, 5-8 F, and 6-10B were kindly provided by the Cancer Center of Sun Yet-Sen University (Guangzhou, China) and preserved in our laboratory. The HNE1 and HNE2 cell lines were purchased from the Cell Center of Xiangya School of Medicine at Central South University (Changsha, China). The embryonic kidney cell lines HEK293 and HEK293FT were purchased from the American Type Culture Collection (ATCC; Manassas, VA, USA). The cells were routinely cultured in Dulbecco’s modified Eagle’s medium (DMEM; Life Technologies, Carlsbad, CA, USA) or Roswell Park Memorial Institute-1640 (RPMI-1640; Life Technologies) medium supplemented with 10% fetal bovine serum (FBS; BI, Jerusalem, Israel), 100 U/mL penicillin, and 100 μg/mL streptomycin at 37 °C in a 5% CO_2_ atmosphere. All cell lines were regularly tested to confirm the absence of mycoplasma infection.

### RNA extraction, reverse transcription, and real-time RT–PCR

Total RNA from 5-8 F and HNE1 cells was isolated with TRIzol reagent (Invitrogen, Carlsbad, USA), and then 1 μg of total RNA was used to synthesize cDNA by using a RevertAid First Strand cDNA Synthesis Kit (K1622; Thermo Scientific, Waltham, USA) according to the instructions. The Platinum SYBR Green qPCR SuperMix-UDG reagents (Accurate Biotechnology, Hunan, China) were used in quantitative RT–PCR analysis with the Bio-Rad CFX96 Touch sequence detection system (Bio–Rad Laboratories Inc.). GAPDH was used as an internal control. The sequences of the primers were as follows: BIRC2 forward primer: 5′-TGGAGATAGGGTAGCCTGCTT-3′, reverse primer: 5′-GGAAAATGCCTCCGGTGTTC-3′; GAPDH forward primer: 5′-TCTGACGTGCCGCCTGGAGA-3′, reverse primer: 5′-CAGCCCCGGCATCGAAGGTG-3′; BRD7 forward primer: 5′-AACGACGTTGGGACTTCTCC-3′, reverse primer: 5′-TGCTCCATTTCTTTTGCTGTGT-3′. The relative fold changes in expression were analyzed using the 2^−ΔΔCT^ method, and each sample was analyzed in triplicate.

### Stable cell lines

The pCDH-copGFP/BRD7 and pCDH-copGFP/BIRC2 recombinant lentiviral expression vectors were constructed by linking the full-length ORF of human BRD7 or BIRC2 with three FLAG tags to the lentivirus expression vector pCDH-copGFP, respectively. To generate NPC cell lines with stable BRD7 or BIRC2 overexpression, the pCDH-copGFP/BRD7 or pCDH-copGFP/BIRC2 vector and the envelope plasmid pMD2.G and packaging plasmid psPAX2 (4:1:3) were first co-transfected into HEK293FT packaging cells in serum-free medium by using Lipofectamine 3000 reagent (Invitrogen, Carlsbad, USA) according to the manufacturer’s protocol. After 48 h, virus particles were collected, filtered, and used for transduction of the target 5-8 F and HNE1 cells. Finally, positive 5-8 F and HNE1 cells were selected via FACS, and NPC cell lines with stable BRD7 or BIRC2 overexpression were obtained.

### RNA interference and transfection

Specific small interfering RNAs (siRNAs) targeting BIRC2/BRD7 were purchased from RiboBio (Guangzhou, China). For transfection, 5 μL of siRNA targeting human BIRC2/BRD7 and 5 μL of Lipofectamine 3000 were diluted in 125 μL of reduced serum medium (Opti-MEM, Invitrogen). The mixtures were incubated for 15 min before being added dropwise to culture wells to achieve a final siRNA concentration of 50 nM.

### CCK-8 and colony formation assays

CCK-8 and colony formation assays were performed as we described previously [44]. For CCK-8 assays, briefly, cells were seeded into 96-well plates at a density of 1000 cells/well, and 10 μL of Cell Counting Kit-8 (CCK-8) reagent (Selleck, Houston, TX, USA) was added per well at the indicated time points. After incubation at 37 °C for 2 h, the number of viable cells was determined by measuring the optical density at 450 nm with a microplate reader (Beckman, Brea, CA, USA). For colony formation assays, 1000 cells per well were seeded into 6-well plates and cultured for 10-14 days. After washing three times with PBS, cells were stained with Crystal Violet Staining Solution (Beyotime, Beijing, China). Next, images of each well were acquired by microscopy, and each colony containing more than 50 cells was counted as one positive colony. All experiments were performed in triplicate.

### Wound healing and Transwell invasion assays

Cells were seeded in 6-well plates. When the cells were approximately 90% confluent, a 10-µL plastic pipette tip was used to create a straight scratch through the cell monolayer; images of the scratch width were acquired at 0, 24, and 48 hours after the initial scratch was made, and the area of cell invasion was measured for evaluation of cell migration. For transwell invasion assays, Transwell chambers (Corning, NY, USA) with a membrane pore size of 8 μm were coated without or with matrigel (BD Biosciences, NY, USA). Subsequently, 3×10^4^ cells suspended in 200 μL of serum-free medium were seeded in the upper chambers, and 600 μL of medium supplemented with 20% FBS was placed in the lower chambers. After culture for 36–48 hours, cells had penetrated the coated membrane to the lower surface, and the cells in the upper chamber were carefully removed with a cotton swab. The cells were fixed with 4% paraformaldehyde and stained with hematoxylin (Beyotime), and the number of migrated cells was counted.

### Luciferase reporter assay

The pGL3 promoter, pGL3 enhancer vector and pRL-TK vector were purchased from Promega (Fitchburg, WI, USA), and the potential promoter sequence of BIRC2 was inserted into the pGL3 enhancer vector (pGL3 enhancer/BIRC2 promoter). As B7BS was confirmed as the binding region of BIRC2 with BRD7 protein, and the nucleotide sequence (GGGGAGGAAG) within B7BS was identified as the potential motif binding to BRD7. Therefore, both of the recombinant reporter vectors fused with the B7BS (pGL3 promoter/B7BS) and mut-B7BS (pGL3 promoter/mut-B7BS) were constructed by using the pGL3 promoter vector. The proper insertions were further confirmed by sequencing. 5-8 F, HNE1 and HEK293 cells with BRD7 overexpression were seeded in triplicate in 24-well plates. When the cells grew to the appropriate density, the recombinant reporter vectors and internal control vector pRL-TK were co-transfected into cells using Lipofectamine 3000 reagent. Cells were harvested 48 h post-transfection. Firefly and renilla luciferase activities were measured using the dual-luciferase reporter assay system (Promega) as previously described [44].

### Chromatin immunoprecipitation (ChIP) assays

ChIP assays were performed using 10 million 5-8 F and HNE1 cells. Cells were crosslinked with 1% formaldehyde for 10 min at 37 °C, and stop-fix solution containing glycine (125 mM) was then added to quench DNA–protein crosslinking for 5 min at room temperature. Then, the cells were harvested by scraping and collected by centrifugation at 1500 rpm for 5 min. After cell lysis and chromatin extraction, chromatin was sheared into fragments of 400-600 bp using a BioRuptor sonicator. The lysate was cleared by centrifugation, and the supernatant was collected as the whole-cell extract (WCE). Next, chromatin was immunoprecipitated with immunoglobulin IgG (Sigma–Aldrich, St. Louis, MO) and an anti-H3K4me1 antibody (Cat# A2355, ABclonal, Wuhan, China) with the Protein A/G Magnetic Beads system (Selleck Chemicals, Houston, TX, USA) according to the manufacturer’s protocols. After overnight incubation with shaking at 4 °C, the antibody–bead complexes were washed sequentially with low salt buffer, high salt buffer, LiCl buffer and TE buffer. Subsequently, crosslinks were reversed by incubation overnight at 65 °C in the presence of proteinase K, and DNA was extracted with an equal volume of a neutral phenol: chloroform: isoamyl alcohol (25: 24: 1) mixture. Finally, the purified DNA was used for subsequent PCR. The primers used for confirmation of the BRD7 binding sites in B7BS via PCR are described in Table 2.

**Table 2:** The primers sequences for ChIP assay.

Primer name	Sequences (5^′^- 3^′^)	Length (bp)
ChIP-B7BS-1-199bp-F1	ACACCTGGCTCATTTTTCTT	20
ChIP-B7BS-1-199bp-R1	AAAATTAGCTGGGTATGGTG	20
ChIP-B7BS-200-386bp-F2	TGTATTTTTAGTAGAGTGGG	20
ChIP-B7BS-200-386bp-R2	TTTGGAACCTGAATGAATTT	20
ChIP-B7BS-387-573bp-F3	CAACCAACTGGCAATAAACTC	21
ChIP-B7BS-387-573bp-R3	GCATGTGTTAAATAATGTTGT	21
ChIP-B7BS-574-755bp-F4	AATAAAATGGGAGAGGGGGTG	21
ChIP-B7BS-574-755bp-R4	AGGACTACAGACTGCCTCTAG	21

### Western blot analysis

Cells were harvested and lysed with cell lysis reagent (NCM Biotech, Suzhou, China), and protein was collected. A total of 20 µg of total protein per lane was separated by 4-15% SDS-polyacrylamide gel electrophoresis and was then transferred to 0.2 μM or 0.45 μM PVDF membranes (Millipore, Billerica, USA). Subsequently, the membranes were blocked with 5% nonfat milk for 60-120 min. They were incubated separately at 4 °C overnight with the following primary antibodies: anti-*BRD7* (Cat# 51009-2-AP, 1:500) and anti-*GAPDH* (Cat# 60004-1-Ig, 1:10,000) (Proteintech Group, Inc., Wuhan, China); anti-*BIRC2* (Cat# 70008, 1:1,000), anti-E-cadherin (Cat# 14472, 1:1000) and anti-Snail (Cat# 3879, 1:1000) (purchased from CST, MA, USA); anti-Flag (Cat# F2555, 1:1000, Sigma-Aldrich, St. Louis, MO); anti-Vimentin (Cat# ARG66199, 1:1000, Arigo, Taiwan, China) and anti-N-cadherin (Cat# AF5239, 1:1000, Affinity Biosciences, Jiangsu, China). The membranes were then incubated with species-matched secondary antibodies for 60 min at 37 °C. Bands were visualized by Western Blotting Substrate (Share-Bio, Shanghai, China), and signals were detected by an enhanced chemiluminescence detection system according to the manufacturer’s protocol (MiniChemi™ I, SAGECREATION, China).

### In vivo nude mouse models

All animal studies were approved by the Institutional Animal Care and Use Committee of Central South University (Changsha, China). The female BALB/c nude mice (ages 4–5 weeks, 18–20 g) used in this experiment were purchased from Hunan Slake Jingda Experimental Animal Co., Ltd. For the tumor growth model, we divided the 20 BALB/c nude mice into four groups: 5-8 F/Ctrl, 5-8 F/BRD7, 5-8 F/BIRC2 and 5-8 F/BRD7 plus BIRC2, and each group contains five mice. A total of 5×10^6^ cells in 0.9% saline solution with Matrigel (total volume of 150 μL) were injected into the axilla of each mouse, and the mice were checked every 2 days. Tumors were measured using a caliper, and volumes were calculated using the following equation: volume = (length×width^2^)/2. Tumor growth curves were plotted. All mice in the four groups were sacrificed 20 days after subcutaneous inoculation, and all tumors were excised, weighed, fixed with 4% formaldehyde and embedded in paraffin for IHC staining.

For the lung metastasis model, age-matched female mice were used as described above, which was also divided into four groups and each group includes five mice. A total of 2×10^6^ 5-8 F/Ctrl, 5-8 F/BRD7, 5-8 F/BIRC2 or 5-8 F/BRD7 plus BIRC2 cells in 0.9% saline solution were injected into the tail veins of nude mice. The mice were housed under constant environmental conditions and were examined and weighed every 4 days. The mice were sacrificed after 8 weeks, and the lungs were removed, photographed, fixed with 4% formaldehyde and embedded in paraffin for hematoxylin and eosin staining. Subsequently, the number of tumor nodules in the lungs of each mouse was determined under a microscope.

### IHC staining

IHC staining was performed as described previously [44]. Sections were incubated with rabbit anti-*BRD7* (1:400), anti-BIRC2 (Cat# 10022-1-AP, 1: 400) and anti-CDK4 (Cat# 11026-1-AP, 1:100) (Proteintech Group, Inc., Wuhan, China), anti-cleaved-PARP (Cat# 5625, 1:100, CST, MA, USA), anti-ki-67 (Cat# BS65573, 1:100, Bioworld, Nanjing, China) antibodies overnight at 4 °C. Immune complexes were visualized using the MaxVision HRP-polymer IHC kit detection system. At least three random fields per sample were examined, and images were acquired using a microscope (Olympus Corporation, Tokyo, Japan). All slices were evaluated by two pathologists without knowledge of the clinical outcomes. The immunostaining intensity was scored as 0 (negative), 1 (weak), 2 (moderate), or 3 (strong), and the proportion of positively stained cells was scored as 0 (<5%), 1 (6–25%), 2 (26–50%), 3 (51–75%), or 4 (>75%). The scores of the molecules of interest in the samples were calculated as the product of the intensity and percentage scores and ranged from 0 to 12. The expression level was classified as low or high with the median total score as the cutoff [44–46].

### Statistical analysis

Each experiment was repeated independently at least three times. All statistical analyses were performed using Prism 6.0 software (San Diego, CA, USA). All data are presented as the mean ± standard deviation (mean ± SD) values. The associations between the expression levels of BRD7 and BIRC2 and clinicopathological characteristics in NPC were assessed using the χ^2^ test. Statistical significance was assessed by performing the student^,^s t-test. *P* values less than 0.05 indicate statistical significance.

## Supplementary information

A reproducibility checklist in this manuscript

supplemental material in this manuscript.

Original Blots in this manuscript.

## Data Availability

All data generated or analyzed during this study are included in this published article and its additional files.
