# Supplementary material for: BRD7 inhibits enhancer activity and expression of BIRC2 to suppress tumor growth and metastasis in nasopharyngeal carcinoma
Source: Cell Death Dis. 2023 Feb 14;14(2):121. doi: 10.1038/s41419-023-05632-3 (PMC9929072; doi:10.1038/s41419-023-05632-3)
Supplement: Supplementary file 2 — supplemental material in this manuscript. [file 41419_2023_5632_MOESM2_ESM.docx]

**Supplementary Data**

**

**

**Figure S1. BIRC2 was identified as a downstream target gene of BRD7 by negatively regulating the activation of the BIRC2 enhancer.** (A) and (B) Western blotting and qRT-PCR assays to detect the protein and mRNA expression of BIRC2 after BRD7 knockdown. GAPDH served as an internal control. C Western blotting to confirm BRD7 and BIRC2 protein levels. GAPDH served as an internal control. D The dual-luciferase reporter assays determined the BIRC2 promoter activity in HEK293 cell. E UCSC website predictions H3K4me1 enrichment at genomic B7BS region. F The dual-luciferase reporter assays determined the B7BS activity in HEK293 cell. The error bars are presented as the mean ± SD. **P*＜0.05, ***P*＜0.01, ****P*＜0.001; NS, no significance. All experiments were performed in triplicate.





**Figure S2. Detection and confirmation of BIRC2 expression.** A Relative level of BIRC2 in nasopharyngeal epithelial cell line (NP69) and NPC cell lines (CNE1, 6-10B, 5-8F, HNE-1, HNE2 and CNE2. (B) and (C) Relative mRNA expression levels of BIRC2 using qPCR. The error bars are presented as the mean ± SD. **P*＜0.05, ***P*＜0.01, ****P*＜0.001, NS, no significance. All experiments were performed in triplicate.


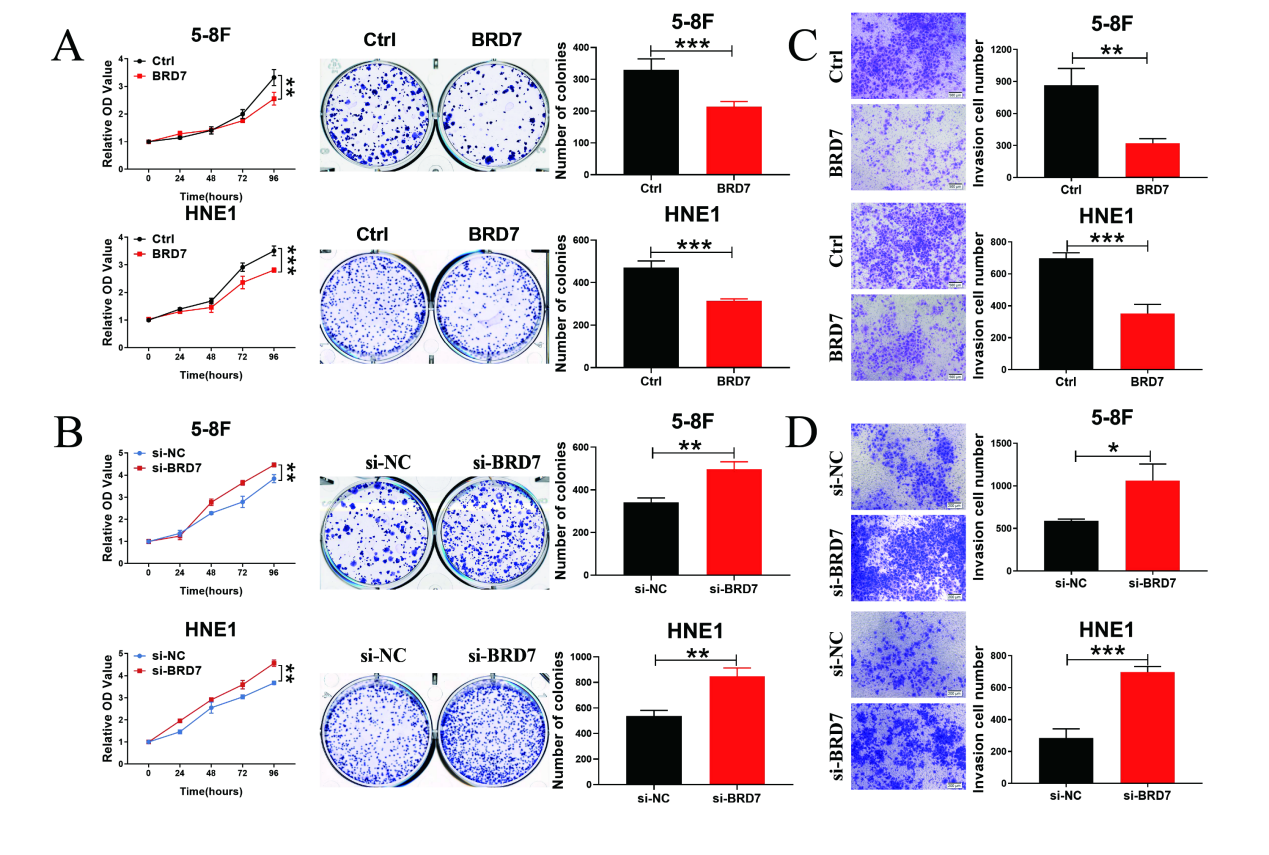


**Figure S3. BRD7 inhibits tumor progression and functions as a tumor suppressor in NPC.** (A) and (B) CCK-8 and colony-forming assays were performed to detect the cell proliferation of 5-8F and HNE1 cells after overexpression or knockdown of BRD7. (C) and (D) Matrigel invasion analysis of cell invasive capability in 5-8F and HNE1 cells after overexpression or knockdown of BRD7. Error bars represent the mean ± SD. *P<0.05, **P<0.01, ***P<0.001. All experiments were performed in triplicate.


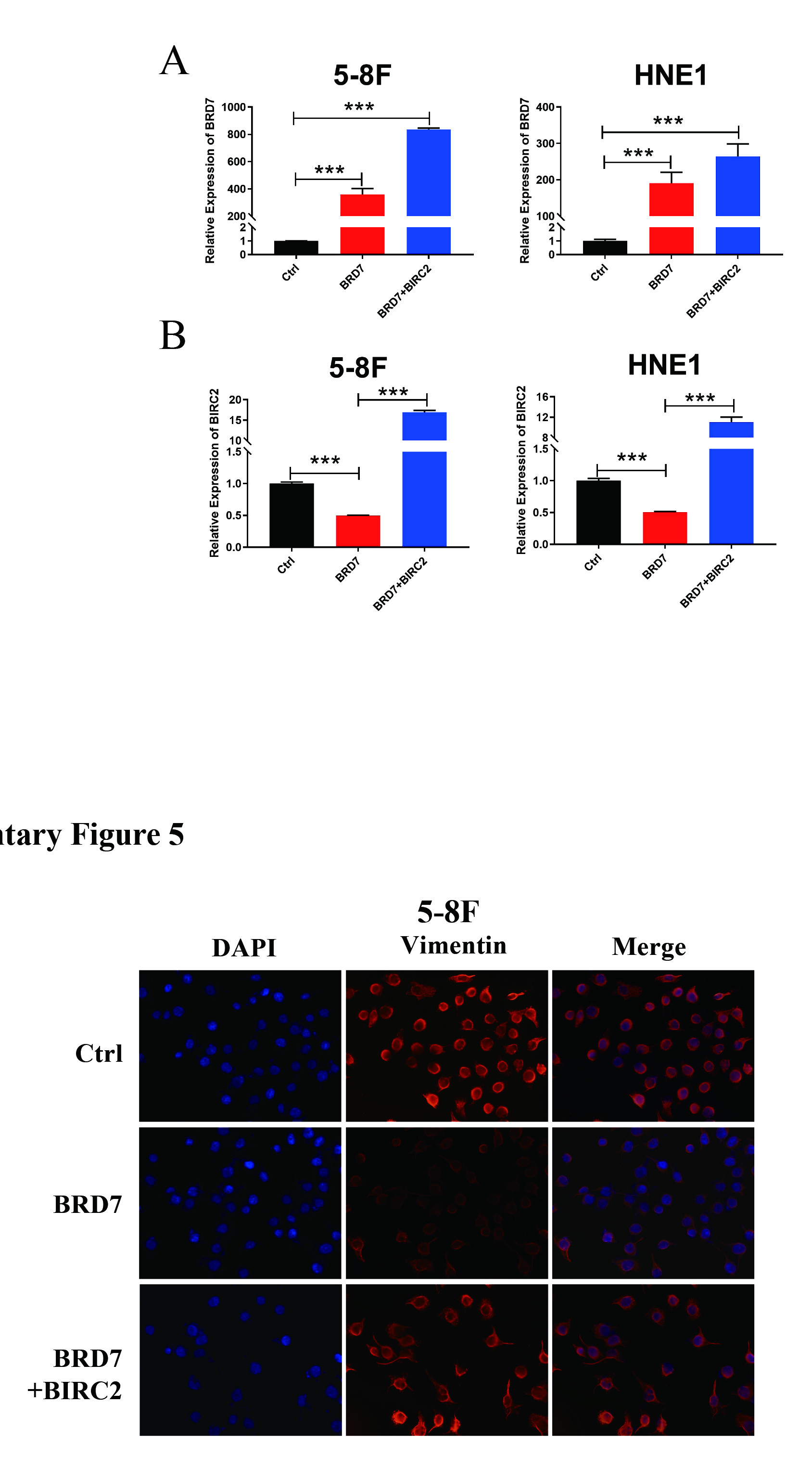


**Figure S4. Detection and confirmation of BIRC2 and BRD7 expression.**  (A) and (B) Relative mRNA expression levels of BIRC2 and BRD7 were detected in 5-8F and HNE1 cell lines using qPCR assay, GAPDH served as an internal control. The error bars are presented as the mean ± SD. ****P*＜0.001, All experiments were performed in triplicate.


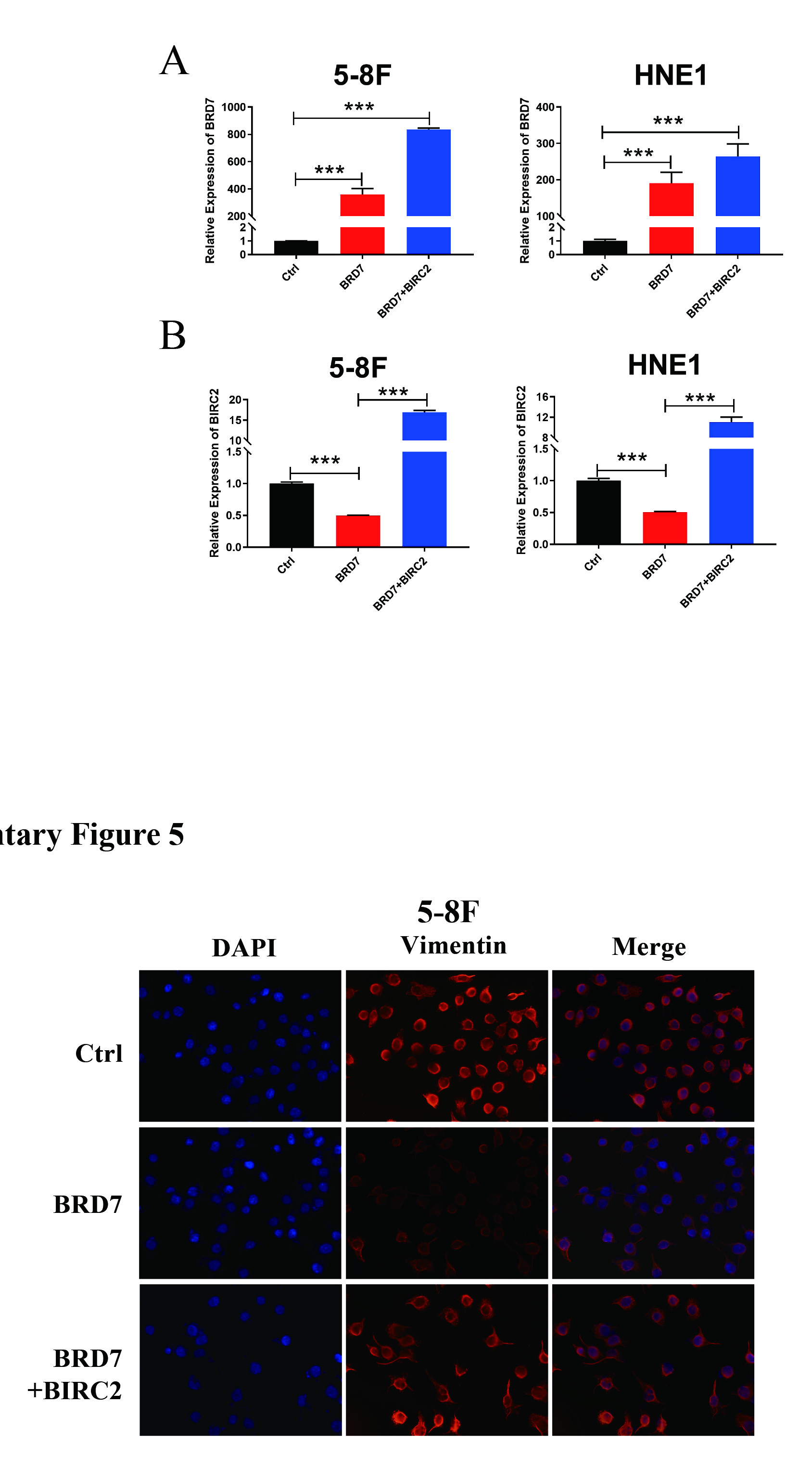


**Figure S5. Detection of subcellular colocalization and expression of Vimentin in 5-8F cell.** Subcellular colocalization and expression of endogenous Vimentin detected by immunofluorescence assay in 5-8F cell. Images were obtained via confocal microscopy.


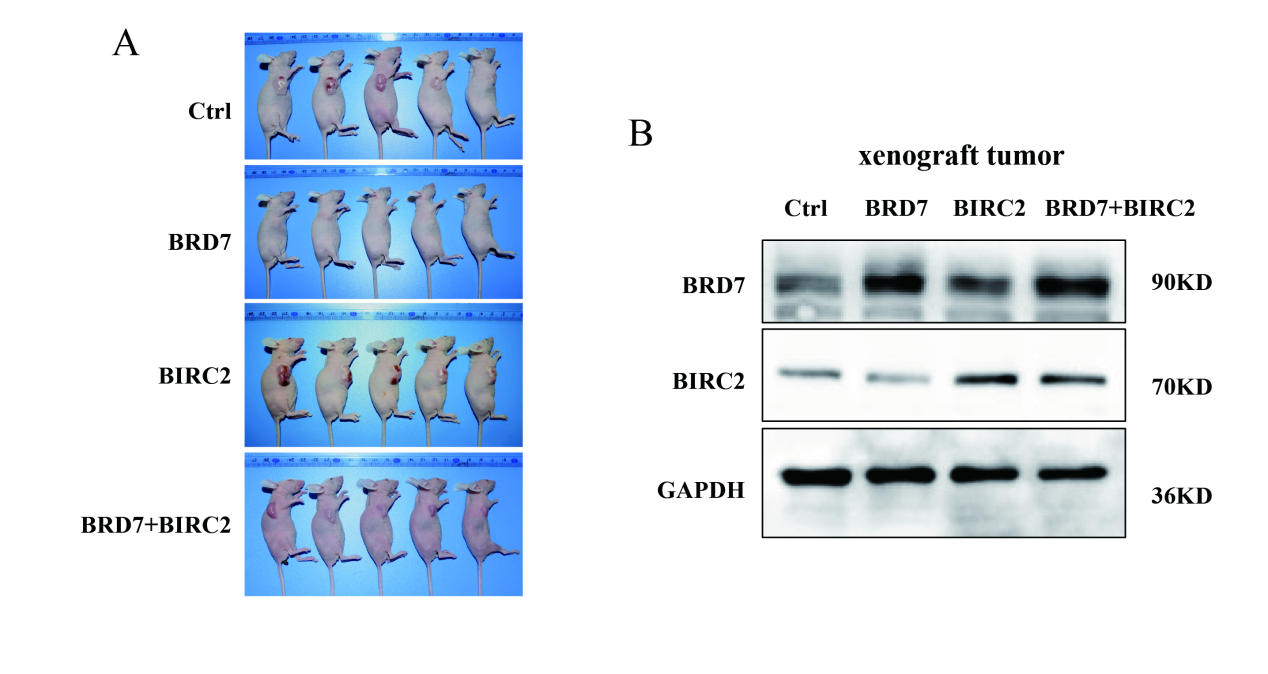


**Figure S6. Effect of BRD7/BIRC2 axis on xenograft tumor growth.** A The photographs of the 5-8F xenograft model in nude mice. B BRD7 and BIRC2 protein levels were confirmed by western blotting assay in xenograft tumor tissues. GAPDH served as an internal control.
