# Supplementary material for: BRD7 inhibits enhancer activity and expression of BIRC2 to suppress tumor growth and metastasis in nasopharyngeal carcinoma
Source: Cell Death Dis. 2023 Feb 14;14(2):121. doi: 10.1038/s41419-023-05632-3 (PMC9929072; doi:10.1038/s41419-023-05632-3)

Original blots in this manuscript

1. Original blots used in Figure 1B

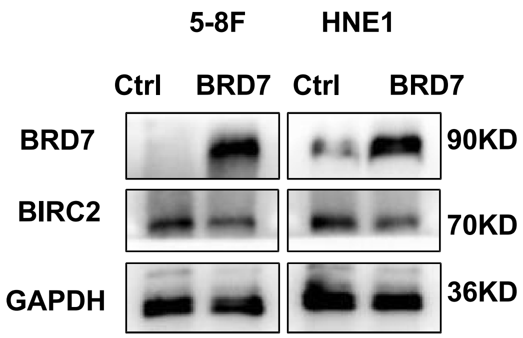

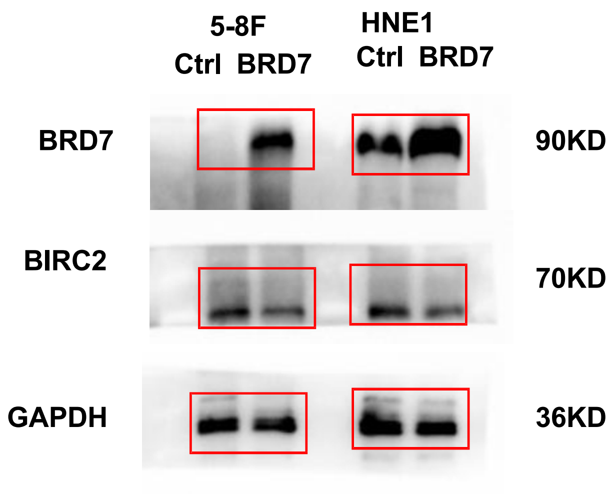

2. Original blots used in Figure 2A

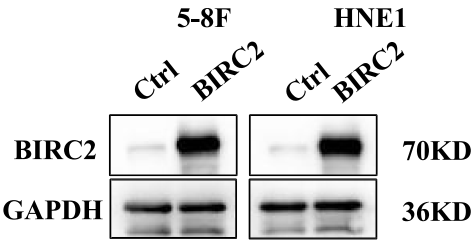

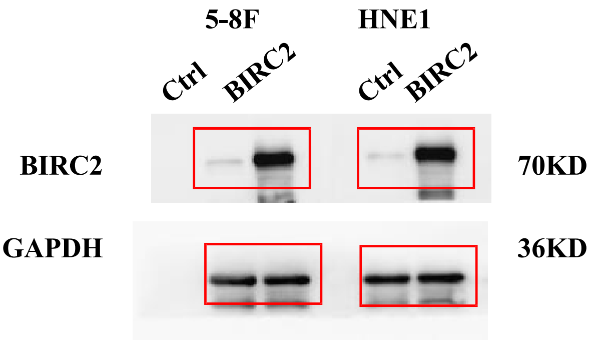

3. Original blots used in Figure 3A

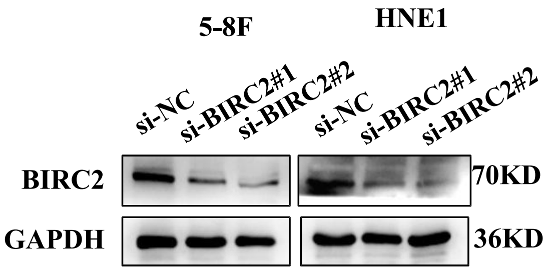

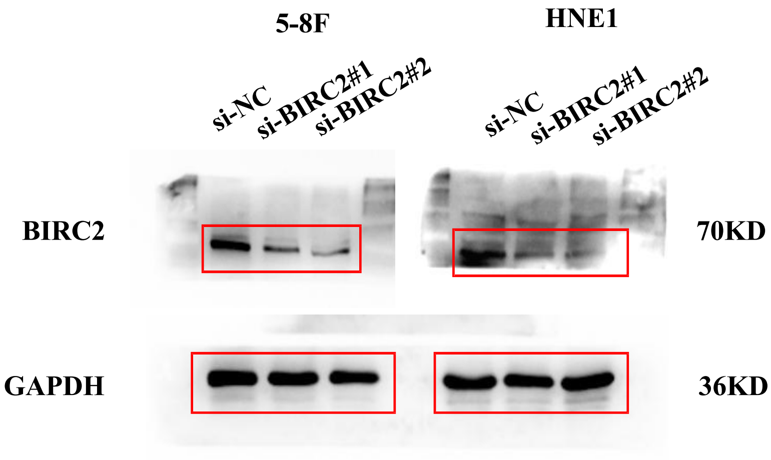

4. Original blots used in Figure 4A

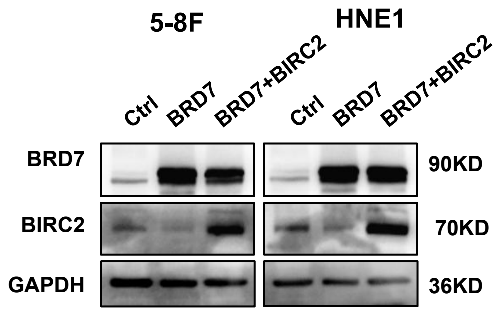

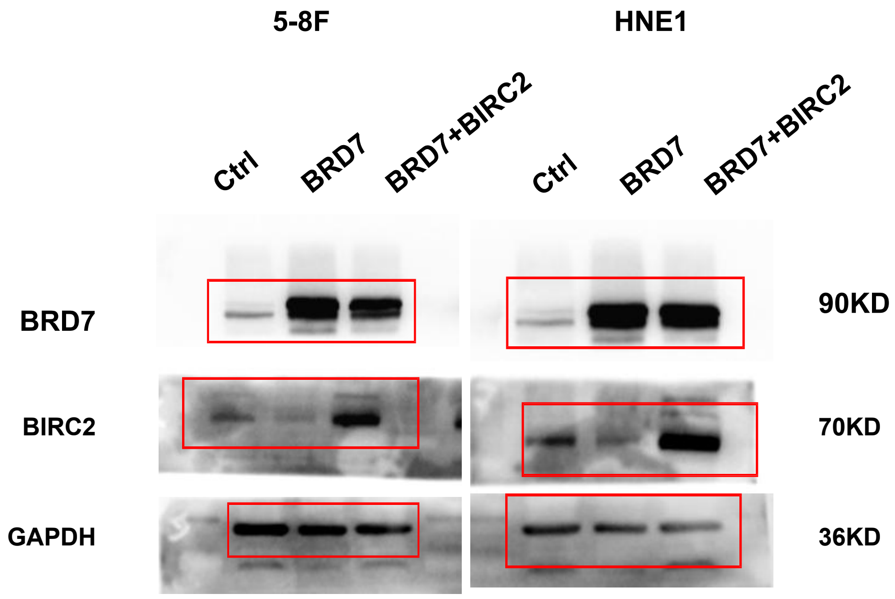

5. Original blots used in Figure 4E

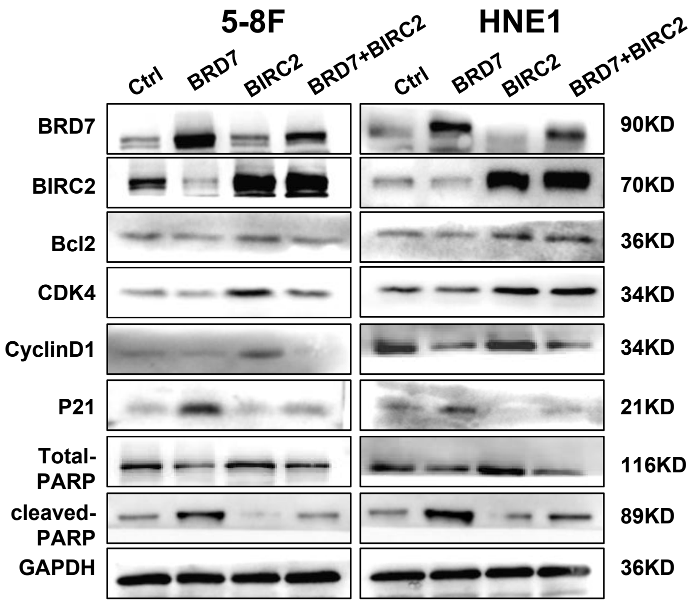

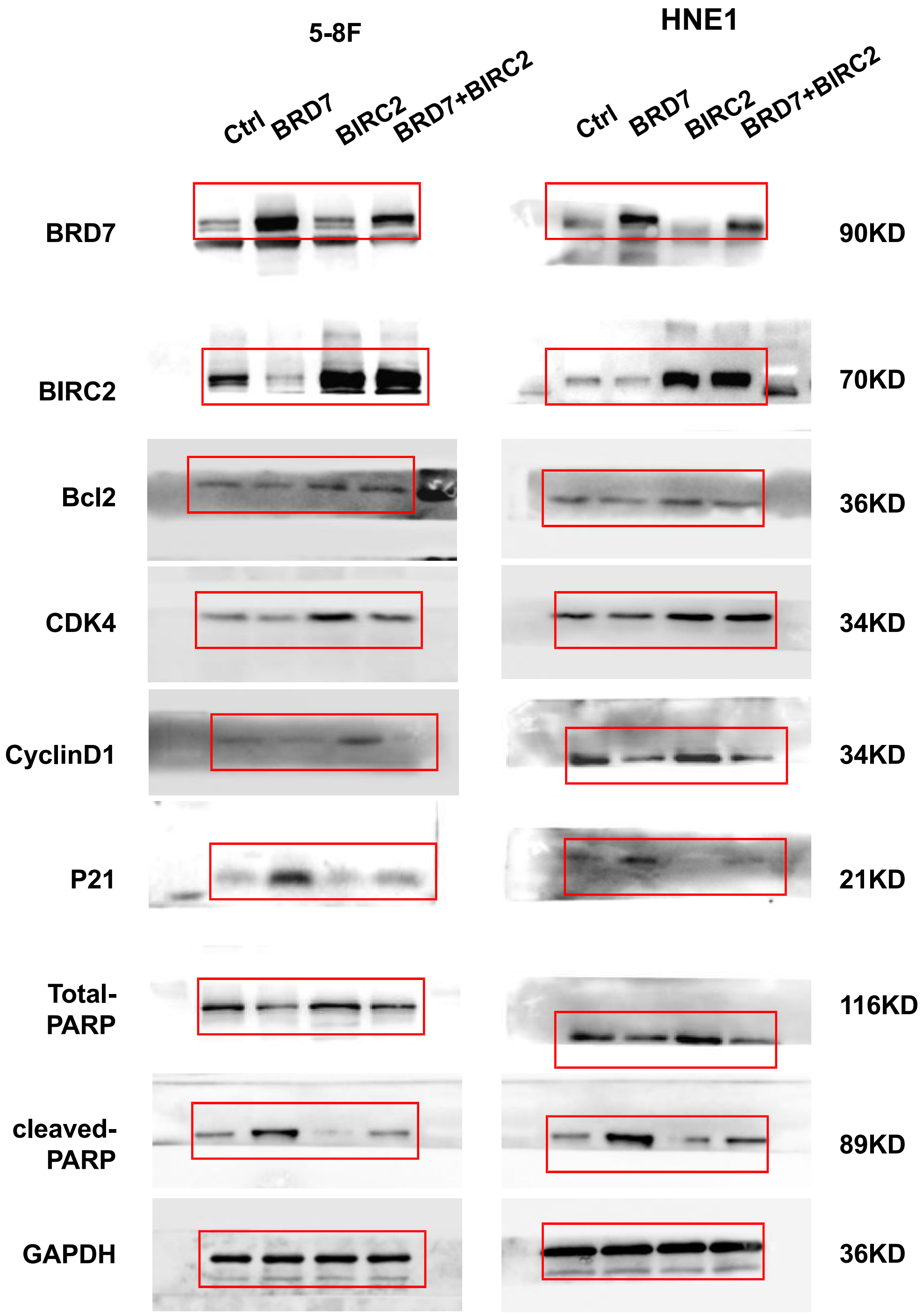

6. Original blots used in Figure 5C

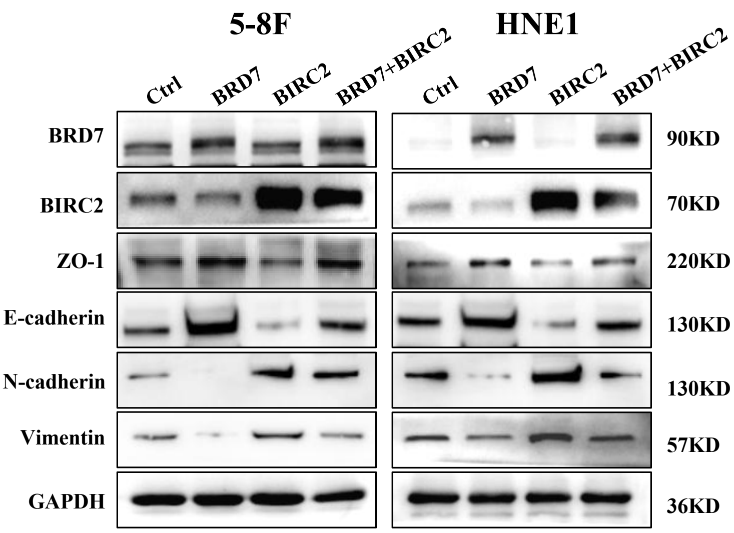

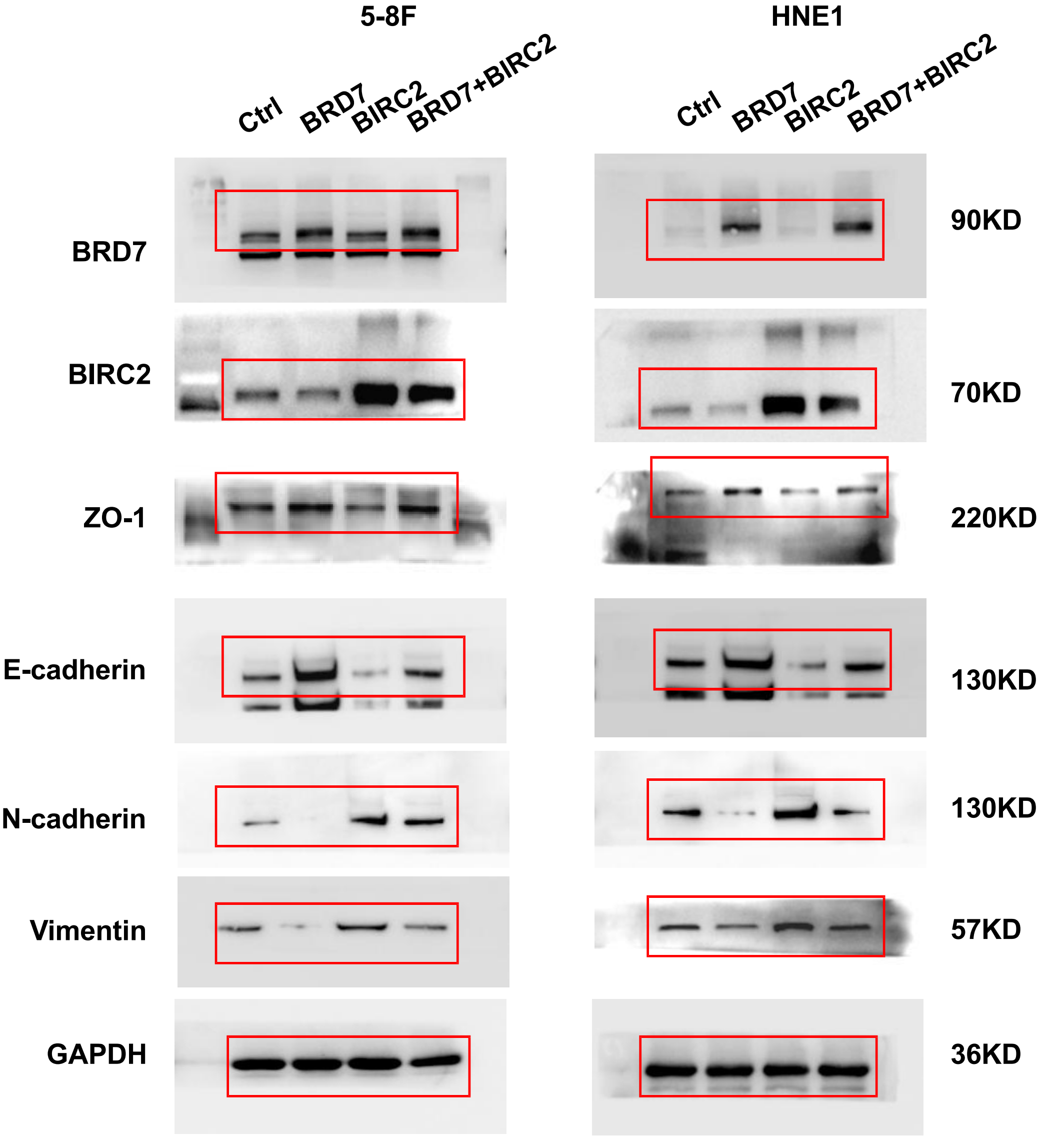

6. Original blots used in Figure S1A

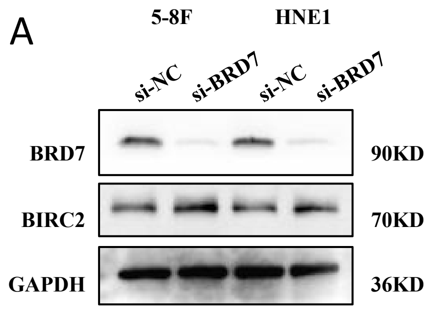

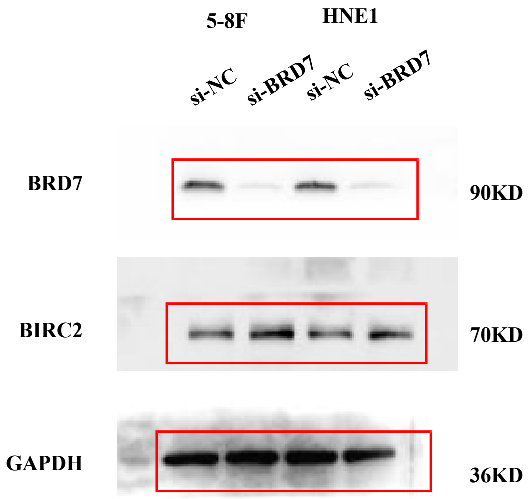

7. Original blots used in Figure S1C

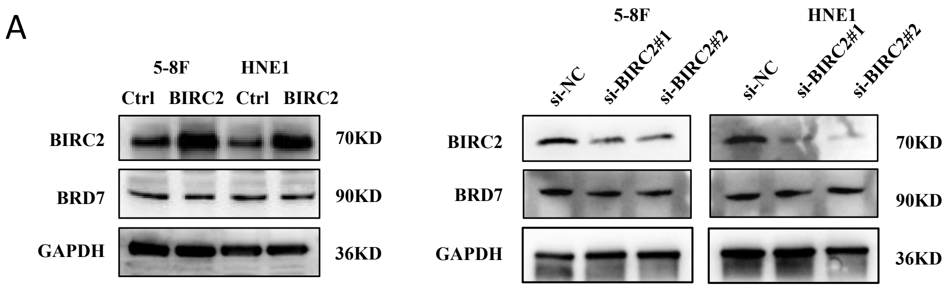

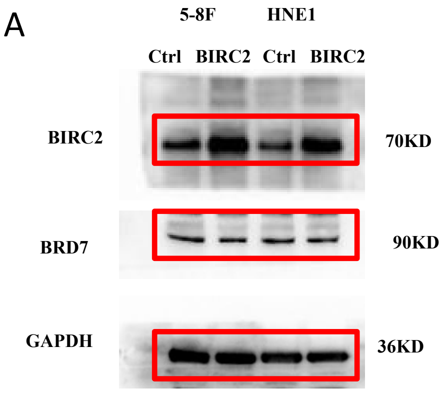

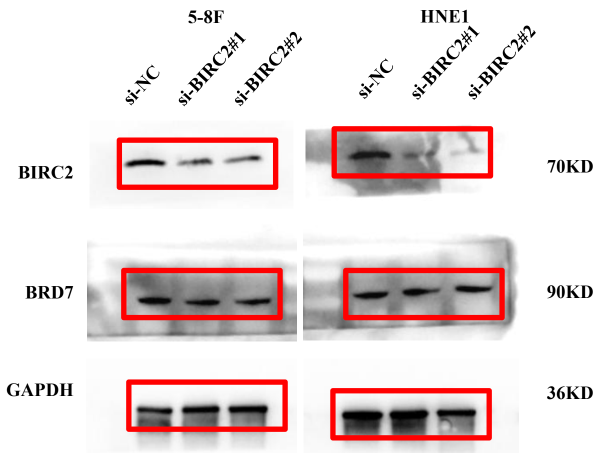

8. Original blots used in Figure S2A

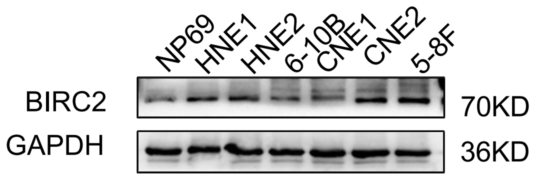

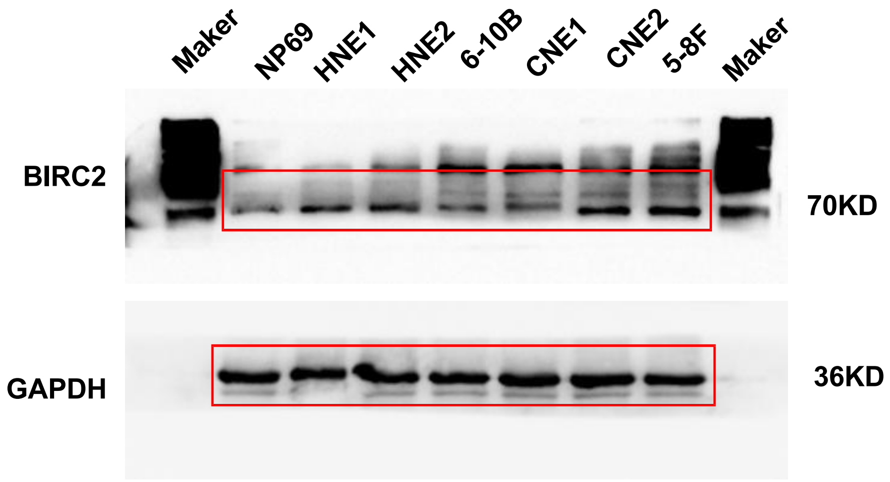

9. Original blots used in Figure S6B

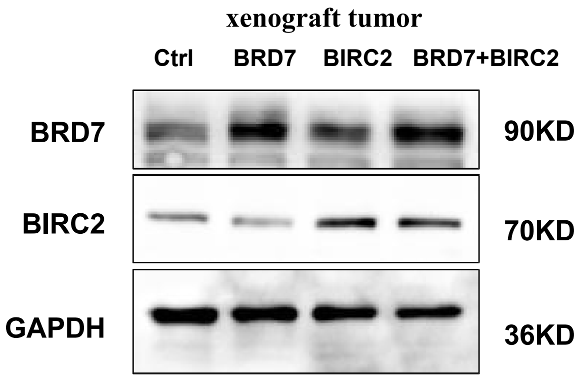

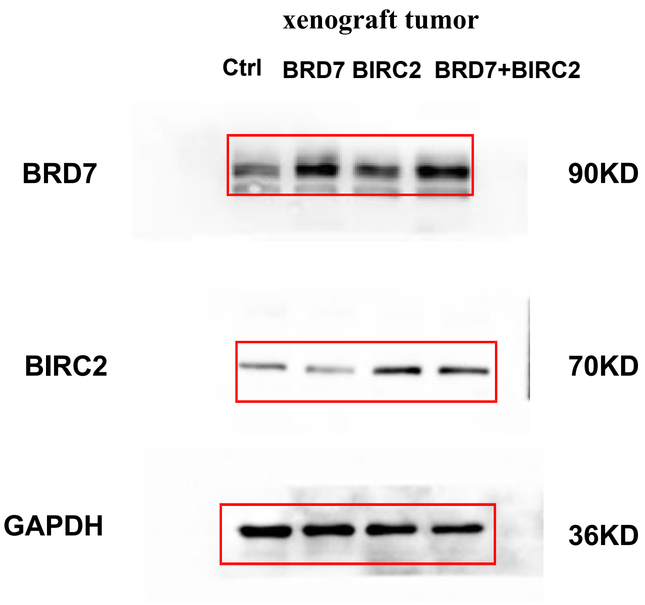

Supplement: Supplementary file 3 — Original Blots in this manuscript. [file 41419_2023_5632_MOESM3_ESM.pdf]
